# Mesenchymal stem cells and extracellular vesicles in therapy against kidney diseases

**DOI:** 10.1186/s13287-021-02289-7

**Published:** 2021-03-31

**Authors:** Yuling Huang, Lina Yang

**Affiliations:** Departments of Geriatrics, The First Affiliated Hospital of China Medical University, 155th Nanjing North Street, Shenyang, 110001 Liaoning China

**Keywords:** Kidney diseases, Mesenchymal stem cells, Extracellular vesicles

## Abstract

Kidney diseases pose a threat to human health due to their rising incidence and fatality rate. In preclinical and clinical studies, it has been acknowledged that mesenchymal stem cells (MSCs) are effective and safe when used to treat kidney diseases. MSCs play their role mainly by secreting trophic factors and delivering extracellular vesicles (EVs). The genetic materials and proteins contained in the MSC-derived EVs (MSC-EVs), as an important means of cellular communication, have become a research focus for targeted therapy of kidney diseases. At present, MSC-EVs have shown evident therapeutic effects on acute kidney injury (AKI), chronic kidney disease (CKD), diabetic nephropathy (DN), and atherosclerotic renovascular disease (ARVD); however, their roles in the transplanted kidney remain controversial. This review summarises the mechanisms by which MSC-EVs treat these diseases in animal models and proposes certain problems, expecting to facilitate corresponding future clinical practice.

## Introduction

Kidney diseases have become an important global health issue as their incidence and fatality rate increase [1]. Common kidney diseases include acute kidney injury (AKI), chronic kidney disease (CKD), diabetic nephropathy (DN), lupus nephritis, and hypertensive nephropathy, induced by various causes. The main therapeutic methods against these kidney diseases include drug therapy, dialysis, and kidney transplantation [2, 3]; however, new therapies emerge due to the limitations of drug therapy, inconvenience of dialysis, and shortage of donors for kidney transplantation [4]. In recent years, stem cells, as a new regenerative therapy, have been used to treat numerous diseases, including kidney diseases [5]. Therefore, MSCs have become a new means of treating kidney diseases. Compared with MSCs, treating kidney diseases with MSC-derived extracellular vesicles (MSC-EVs) is characterised by advantages such as lower immunogenicity and tumorigenicity [6]; however, the pathway and mechanism of action of MSC-EVs in treating kidney diseases have not been elucidated, and the clinical use of MSC-EVs is still being explored. Considering this, in this review, we summarise the status of research involving MSC-EVs in the treatment of kidney diseases.

## MSC-EVs

### Biological characteristics of MSCs

**Fig. 1:**
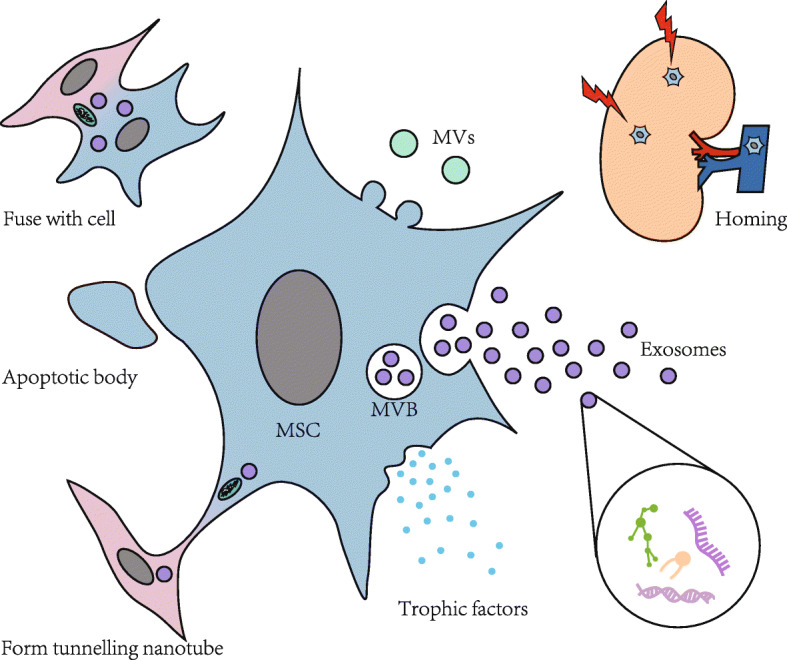
The function of MSCs/MSC-EVs. MSCs can be specifically located in the injured zones of the kidney. Then, MSCs act on peripheral cells by secreting trophic factors such as growth factors, chemokines, and cytokines, or deliver subcellular structures and even mitochondria by forming tunnelling nanotubes, secreting EVs, and fusing with cells. EVs enter endosomes to form MVBs by way of budding, then MVBs are combined with cytomembranes to release EVs. EVs can be divided into exosomes, MVs, and apoptotic bodies. EVs, specially Exos, contain DNA, RNA, proteins, and lipid. MSCs, mesenchymal stem cells; EVs, extracellular vesicles; MVBs, multivesicular bodies; MVs, microvesicles

Stem cells can be divided into two categories: embryonic and adult stem cells, according to their stage of development. Adult stem cells refer to the undifferentiated cells in differentiated tissues and are present in various tissues and organs of a body. MSCs, as a type of self-renewing multipotent adult stem cell, can be differentiated into diverse types of cells. MSCs can be isolated from numerous tissues such as bone marrow-derived MSCs (BMMSCs) [7], adipose-derived MSCs (ADMSCs) [8], human umbilical cord-derived MSCs (huMSCs) [9], human placenta-derived MSCs [10], and those from the dental pulp, skin, blood, and urine [5, 11]. In the existing research, BMMSCs, ADMSCs, and huMSCs are mainly used. Researches have shown that MSCs locate to injured areas via direct interaction and the paracrine effect [12, 13], while being less dependent on the differentiation function [14]. Through marking, it is found that, after being injected into the body, MSCs can be specifically located in the injured zones of the kidney [15–17]. The molecular mechanisms of MSC homing are based on a multistep model, including initial tethering by selectins, activation by cytokines, arrest by integrins, diapedesis or transmigration, and migration toward chemokines [18]. It should be noted that chemokines and their receptors are recognised as important mediators of MSC homing; however, low expression levels of homing molecules limit the efficacy of MSC therapy [19]. de Witte et al. and Schrepfer et al. have described that the main problem after MSC administration is that they do no target the target tissue, limiting their therapeutic effect [20, 21]. Then, target administration, magnetic guidance, genetic modification, and cell surface engineering, among others, are studied to facilitate MSC homing [18]. MSCs are administered to target tissues mainly by systemic delivery and local delivery, the former including intra-arterial and intra-venous and the latter including topical, intra-muscular, direct tissue injection, and catheter-based direct implantation. However, there is no consensus on the optimal method for MSC infusion [12]. Also, MSCs can act on peripheral cells by secreting trophic factors such as growth factors, chemokines, and cytokines or deliver subcellular structures and even mitochondria by forming tunnelling nanotubes, secreting extracellular vesicles (EVs) and fusing with cells [22]. Therein, the synthesis and release of EVs that contain proteins, messenger ribose nucleic acid (mRNA), and micro-ribose nucleic acid (miRNA) through paracrine have become the focus of current research. In short, MSCs/MSC-EVs can function in different ways, as shown in Fig. 1.

### Biological characteristics of EVs

EVs, a type of nanoscale vesicles encapsulated by cytomembranes, can be divided into exosomes (Exos), microvesicles (MVs), and apoptotic bodies (Fig. 1), whose diameters are 30–150 nm, 200–1000 nm, and 800–5000 nm, respectively [23]. EVs, as an important means of intercellular communication, are widely present in the body fluids, including blood, urine, and amniotic fluid. EVs enter endosomes to form multivesicular bodies (MVBs) by way of budding, then MVBs are combined with cytomembranes to release EVs [24]. EVs contain DNA, RNA, proteins, and lipid, and the substances contained in EVs are specifically determined by metrocytes [25]. This lays the foundation for the use of EVs as a diagnostic marker of disease. As a non-invasive diagnostic marker, EVs have attracted much attention. For example, miRNAs of urinary Exos reliably reflect the progression of AKI [26]. EVs are used to characterise the rejection in allogeneic heart transplantation [27]. MSC-EVs can delay the progression of kidney diseases through mechanisms of anti-apoptosis, anti-inflammation, anti-fibrosis, antioxidation, etc. Other research also found that EVs derived from other sources can also improve renal function, including renal tubular cells [28, 29], glomerular MSCs [30], MSCs isolated from testis [31], and even urine [32]. These EVs ushered in a new way of treating kidney diseases.

Moreover, EVs can play a wider spectrum of roles under bioengineering design and control. EVs can carry many types of drugs via pre- and post-release modification [33] and play their therapeutic role by loading substances thereon and subsequent targeting [34], such as micro-molecules, proteins, and nucleic acids [35]. EVs have become a research focus as a drug carrier for various diseases, such as carrying antineoplastic [36] and anti-inflammatory [37] drugs. The specificity of uptake of EVs is highly dependent on the surface of EVs and acceptor cells, including integrins, proteoglycans, lectins, glycolipid, and others, which can aid in targeting [38]. However, the low retention and poor stability of EVs post-transplantation limit further application in clinic practice. To enhance the therapeutic effect of MSC-EVs for kidney diseases, EVs are encapsulated in a collagen matrix [39], matrix metalloproteinase-2-sensitive self-assembling peptide hydrogel [40], and arginine-glycine-aspartate (RGD) hydrogel [41] to prolong their retention and therefore induce sustained release. Demonstrably, RGD hydrogels interact with EVs mediating by integrin subunits αv, β3, and β8 [41]. Besides slowing the eliminating process of EVs, encapsulation can assist EVs kidney to attenuate kidney injury by pathological damage reduction, promotion of cell proliferation, inhibition of renal cell apoptosis, amplification of autophagic activation, and enhancing of angiogenesis as well as ameliorating fibrosis [39–41]. It is also found the overexpression of Oct-4 can improve the therapeutic effect of MSC-EVs [42], and hypoxia stimulates the MSCs to secrete more EVs [43]. Erythropoietin-processed MSC-EVs can increase the miRNA content in EVs and therefore may help to enhance the protective effect on the kidney [44].

## MSC-EVs and kidney diseases

### MSC-EVs and AKI

AKI is prevalent in critically ill patients, even the mortality of those AKI patients not in intensive care units is as high as 10–20% [45]. There is still a lack of specific and effective therapies for AKI, while stem cell transplantation is promising. Numerous experiments have confirmed the benefits of MSCs in treating AKI, and many methods of enhancing the effect of MSCs have emerged in recent years. For example, IL-17A is found able to increase the percentage of Treg via the COX-2/PGE2 pathway and simulate the immunosuppression function of MSCs [46]; by coating MSCs with antibodies directed against kidney injury molecule-1, the retention of MSCs in ischaemic kidney is prolonged [47]; in the mouse model of cisplatin-induced AKI, MSCs are injected directly to the aorta using a minimally invasive technique, which improves the effective rate of utilisation of MSCs [48].

**Fig. 2:**
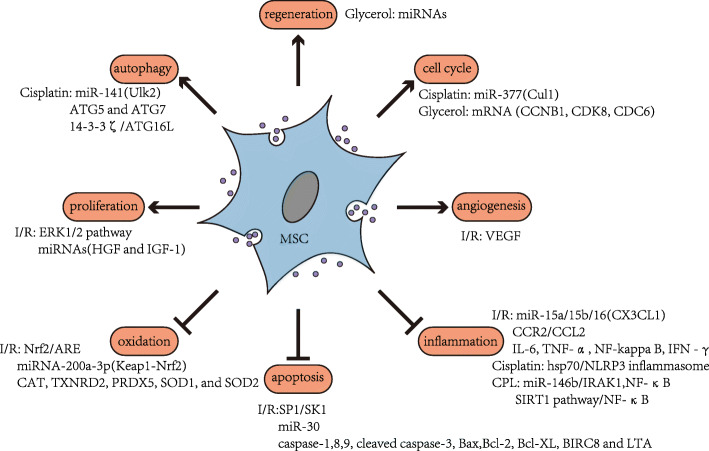
Functional pathways of MSC-EVs in different AKI models. MSC-EVs can relieve AKI by inhibiting oxidation, apoptosis, and inflammation and regulating angiogenesis, cell cycle, regeneration, autophagy, and proliferation. MSCs, mesenchymal stem cells; EVs, extracellular vesicles; AKI, acute kidney injury; I/R, ischaemia-reperfusion; CLP, caecal ligation and puncture

As the research progresses, evidence shows that MSC-EVs play a major role in treating AKI. MSC-EVs can relieve AKI by inhibiting oxidation, apoptosis, and inflammation and regulating angiogenesis, cell cycle, regeneration, autophagy, and proliferation [49–54] (Fig. 2). However, for AKIs with different pathogeneses, the signal substances transferred from MSC-EVs to the target cells exhibit their unique characteristics. The main pathogeneses of AKI include renal toxicity of drugs, ischaemic-reperfusion injury (IRI) caused by transplantation, and sepsis. Correspondingly, experimental AKI models are mainly induced by cisplatin, gentamicin, paraquat, ischaemia-reperfusion (I/R) by occlusion of the unilateral or bilateral renal arteries, and sepsis caused by caecal ligation and puncture (CLP). The mechanisms of MSC-EVs in different AKI models in this review are summarised in Table 1.

**Table 1:** Functional pathways of MSC-EVs in different AKI models

Model	Animal	In vitro model	Injection	EVs/MVs/EXO of source	Pathway/key substance	Target	Mechanism	Reference
I/R	Rat	TECs	Intravenous administration	huMSC-EVs	VEGF		Pro-angiogenesis	[49]
	Rat		Caudal vein	hWJMSC-MVs	miR-15a/15b/16	CX3CL1	Anti-inflammation	[55]
	Mouse		Capsule injection	BMMSC-Exo	CCR2	CCL2	Anti-inflammation	[56]
	SD rat	TECs	Caudal vein	hWJMSC-EVs	miR-30	Mitochondria	Anti-apoptosis	[57]
	SD rat			MSC-Exo	IL-6, TNF-α, NF-kappa B, IFN -γ;caspase-9, cleaved caspase-3, Bax, and Bcl-2		Anti-inflammation and anti-apoptosis	[50]
	Rat	NRK-52E cell	Caudal vein	hWJMSC-EVs	Nrf2	ARE	Antioxidation	[57]
	Mouse	TECs	Intravenous administration	hpMSC-EVs	miRNA-200a-3p	Keap1-Nrf2	Antioxidation	[51]
Cisplatin	SCID mouse	TECs	Caudal vein	BMMSC-MVs	Bcl-XL, Bcl2, and BIRC8↑, Casp1, Casp8, and LTA↓		Anti-apoptosis	[58]
	SD rat	NRK-52E cell	Capsule injection	huMSC-Exo	ERK1/2 pathway		Pro-proliferation	[52]
	C57BL/6 mouse	TECs	Intravenous administration	ADMSC-MVs	miR-141	Ulk2	Regulating autophagy	[53]
					miR-377	Cul1	Regulating cell cycle	
	SD rat	NRK-52E cell	Capsule injection	huMSC-Exo		ATG5 and ATG7↑	Pro-autophagy	[59]
	SD rat	HK-2	Capsule injection	huMSC-Exo	14–3-3ζ	ATG16L	Pro-autophagy	[60, 61]
	CD1 mouse	HEK cell		BMMSC-EVs	hsp70↓	NLRP3 inflammasome↓	Anti-inflammation	[62]
Glycerol	SCID mouse	mTECs	Intravenous administration	BMMSC-EVs	mRNA (CCNB1, CDK8, CDC6)		Regulating cell cycle	[63]
					miRNAs	HGF and IGF-1	Pro-proliferation	
	SCID mouse	mTECs	Intravenous administration	BMMSC-EVs	miRNAs		Pro-regeneration	[54]
CPL	Mouse		Caudal vein	huMSC-Exo	miR-146b↑	IRAK1↓, NF-κB↓	Anti-inflammation	[64]
	C57/BL6 mouse		Caudal vein	ADMSC-Exo	SIRT1 pathway	NF-κB	Anti-inflammation	[65]

*MSCs* mesenchymal stem cells, *EVs* extracellular vesicles, *AKI* acute kidney injury, *MVs*: microvesicles, *Exos* exosomes, *I/R* reperfusion, *CLP* caecal ligation and puncture, *BMMSCs* bone marrow-derived MSCs, *ADMSCs* adipose-derived MSCs, *huMSCs* human umbilical cord-derived MSCs, *hWJMSC* human Wharton’s jelly MSCs, *TECs* tubular epithelial cells, *ERK* extracellular regulated kinase

#### I/R-induced kidney injury

I/R is a common pathogenesis of AKI. In animal trials, I/R models are generally established by occluding the unilateral or bilateral renal arteries and veins and then providing oxygen supply. Previous research indicated that huMSC-EVs can alleviate renal IRI in rats independent of the effect of promoting angiogenesis induced by the hypoxia-inducible factor-1 [49]. MSC-EVs can also inhibit macrophages in the I/R model via various pathways to relieve AKI. In the experiments performed by Zou et al., MVs derived from human Wharton’s jelly MSCs (hWJMSCs) suppress the expression of the renal chemokine CX3CL1 by miR-15a/15b/16 and reduce the number of CD68+ macrophages [55]. Shen et al. found that the C-C chemokine receptor-2 expressed on BMMSC-Exos inhibits the recruitment and activation of CCL2 for macrophages by acting as a decoy to bind ligand CCL2 [56].

Apoptosis is closely related to IRI. Gu et al. verified, through in vivo and in vitro experiments, that EVs derived from hWJMSCs (hWJMSC-EVs) decrease apoptosis of renal tubular epithelial cells (TECs) by inhibiting mitochondria fission using miR-30 [57]. Moreover, Li et al. stated that MSC-Exo slowed the progression of IRI by inhibiting expressions of inflammatory factors (IL-6, TNF-α, NF-kappa B, and IFN-γ) and apoptosis-related factors (caspase-9, cleaved caspase-3, Bax, and Bcl-2) [50].

Antioxidation is an effective measure to alleviate I/R. Zhang et al. revealed that hWJMSC-EVs play their antioxidative effect by activating the nuclear factor-erythroid 2-related factor Nrf2/ARE [57]. Thereafter, experiments by Cao et al. demonstrated that BMMSC-EVs activate the Keap1-Nrf2 signalling pathway in the TECs by transferring miRNA-200a-3p, thus modulating the mitochondria to play an antioxidative role [51].

#### Cisplatin-induced AKI model

Models of AKI induced by drugs are generally established through the induction of cisplatin. Using the cisplatin-induced AKI model, Bruno et al. found that BMMSC-MVs protect the kidney by inducing expressions of anti-apoptotic genes (Bcl-XL, Bcl2, and BIRC8) in TECs and inhibiting expressions of pro-apoptotic genes (Casp1, Casp8, and LTA) [58]. Zhou et al. concluded that huMSC-Exos can stimulate proliferation of nephrocytes in vivo and in vitro by inducing the phosphorylation and activation of extracellular regulated kinase (ERK) 1/2 pathway [52]. de Almeida et al. highlighted the function of ADMSC-MVs in regulating injured cells and the specific miRNA-mRNA network. For example, miR-141 targets Ulk2 to regulate autophagy and miR-377 targets Cul1 to modulate the cell cycle [53]. Wang et al. discovered that huMSC-Exo pre-processing can prevent cisplatin-induced renal toxicity in vivo and in vitro by activating autophagy [59]. Jia et al. conducted two studies and identified 14-3-3ζ as a new mechanism of autophagy activated by huMSC-Exos:14-3-3ζ acts on ATG16L, which activates autophagy and therefore prevents cisplatin-induced AKI [60, 61]. Ullah et al. recently proposed that BMMSC-EVs and pulsed focused ultrasound both alleviate cisplatin-induced cell injury by inhibiting hsp70-mediated NLRP3 inflammasomes [62].

#### AKI model due to myolysis induced by glycerinum

In recent years, the AKI model due to myolysis induced by glycerinum also has received much attention. In such a model, Bruno et al. found that BMMSC-EVs (mainly Exos) are enriched in specific mRNA (CCNB1, CDK8, and CDC6), which influence the onset and progression of cell cycles. The enriched miRNAs promote proliferation by growth factors (HGF and IGF-1) and therefore relieve AKI [63]. Through bioengineering, Tapparo et al. increased specific miRNAs (hsa-miR-10a-5p, hsa-miR-29a-3p, hsa-miR-127-3p, and hsa-miR-486-5p) in BMMSC-EVs to simulate the pro-regenerative effect thereof and alleviate the kidney injury induced by glycerinum [54].

#### CLP

The AKI model prepared by CLP simulates the sepsis-related AKI of critically ill patients. In mice with sepsis, Zhang et al. revealed that huMSC-Exos inhibit NF-κB activity by upregulating miR-146b level while downregulating interleukin-1 receptor-associated kinase expression [64]. Similarly, Gao et al. stated that ADMSC-Exo can regulate NF-κB via the SIRT1 signalling pathway, thus inhibiting inflammation of sepsis-related AKI [65].

### MSC-EVs and CKD

There is new evidence proving that, in many cases, AKI may evolve into CKD [66]. After developing AKI, the additional risks of contracting end-stage renal diseases and CKD were estimated to be increased by 0.4 and 10 cases annually in every 100 AKI patients, respectively [67]. CKD is characterised by progressive irreversible fibrosis of the renal parenchyma. Many diseases can evolve into CKD, including AKI, diabetes, atherosclerosis, and nephrotic syndromes. Numerous evidences have been obtained in relation to the treatment of CKD with MSCs in pre- and post-clinical trials. It has been found in recent research that melatonin preconditioning enhances the treatment ability of MSCs in autologous and allogeneic transplantation [68, 69]. In a clinical trial involving an 18-month follow-up of seven eligible CKD patients, the single-dose autologous MSCs have been proven to be safe and tolerable in CKD patients [70]. Researchers then found that the conditioned medium of huMSCs relieves the fibrosis induced by unilateral ureteral obstruction (UUO) by pro-proliferation and anti-apoptosis [71]. To date, many preclinical studies have proven that MSC-EVs are effective in treating CKD.

In the mouse model of chronic renal toxicity due to cyclosporine, the conditioned medium depleted of EVs, MSC-EVs, and EVs can improve the prognosis of kidney diseases [72]. In the aristolochic acid nephropathy model, MSC-EVs significantly reduce expressions of pro-fibrogenic genes such as α-SMA, TGFβ1, and Col1a1 [73]. In the UUO model, Wang et al. found that BMMSC-Exos alleviate renal interstitial fibrosis by inhibiting TGF-β1 with miRNA-let7c [74]. Recently, some researchers found (in the mouse model) that huMSC-Exos relieve renal interstitial fibrosis by suppressing the ROS-mediated P38MAPK/ERK pathway [75]. Chen et al. proposed that the glial-derived neurotrophic factor-modified ADMSC-Exos stimulate the perivascular capillaries in tubulointerstitial fibrosis by activating the SIRT1/eNOS pathway [76]. In addition, previous research also suggested that ADMSC-Exos upregulate the expression of the transcription factor Sox9 of TECs, and the offspring of Sox9^+^ cells facilitate regeneration of renal tubules rather than fibrotic transformation, thus slowing the AKI-CKD transition [17, 77].

#### MSC-EVs and DN

DN is the main pathogenesis for ESRD. There are numerous investigations evincing that MSC transplantation can slow the progression of DN. A randomised controlled trial reported that it is safe and feasible to use the mesenchymal precursor cells in subjects of type 2 diabetes [78]; however, there are immune rejection problems in allogeneic transplantation and damage induced by hyperglycaemia to autologous MSCs. To solve these problems, Nagaishi et al. innovatively used Wharton’s jelly extract supernatant to improve the morphologies, proliferation capacity, and cellular mobilisation capacity of diabetes-derived BMMSCs, which enables effective autologous transplantation [79]. Recently, some researchers also attempted to co-culture MSCs with peritoneal macrophages [80] and to modify MSCs with angiotensin-converting enzyme 2 [81] to improve the treatment capacity of MSCs for DN.

**Fig. 3:**
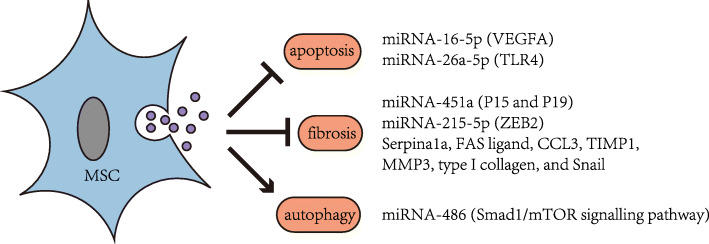
Functional pathways of MSC-EVs in DN. MSC-EVs are able to protect podocytes and other cells by diverse means, including anti-apoptosis, anti-fibrosis, and pro-autophagic effects, thus treating DN. MSCs, mesenchymal stem cells; EVs, extracellular vesicles; DN, diabetic nephropathy

The mechanism of MSC-EVs, as a new means for treating DN, is under constant exploration. Gallo et al. found that MSC/human liver stem cell (HLSC)-EVs can protect mesangial cells from damages induced by hyperglycaemia through the transfer of miR-222 [82]. Also, hyperglycaemia can directly induce the injury of podocytes. The pathological changes of podocytes are closely related to the progression of DN. MSC-EVs are able to protect podocytes and other renal cells by diverse means, including anti-apoptosis, anti-fibrosis, and pro-autophagic effects, thus treating DN (Fig. 3). Duan et al. revealed that the Exo isolated from the conditioned medium of human urine-derived stem cells inhibits the expression of VEGFA and the apoptosis of podocytes by miRNA-16-5p, thereby relieving DN [83]. It is proven by Duan et al. that ADSC-EV miRNA-26a-5p suppresses the hyperglycaemia-induced apoptosis of podocytes in mice by downregulating the TLR4 and NF-κB/VEGFA signalling pathways [84]. Anti-fibrosis is also a major mechanism invoked in DN treatment with MSC-EVs. Zhong et al. reported that MSC-MVs are capable of suppressing cell cycle inhibitors P15 and P19 in vivo and in vitro via miRNA-451a, restarting the cell cycle, and thus reversing the EMT and interstitial fibrosis [85]. Grange et al. considered that EVs of HLSCs and MSCs can inhibit and reverse the progression of glomerular and tubule-interstitial fibrosis in the DN mouse models by downregulating fibrosis-related gene Serpia1a, the FAS ligand, CCL3, TIMP1, MMP3, type I collagen, and Snail [86]. Jin et al. verified that the ADMSC-Exo weakens the EMT of podocytes by suppressing the genetic transcription of ZEB2 by miRNA-215-5p [87]. Autophagy has also been recently considered as a mechanism to delay DN. Ebrahim et al. confirmed that MSC-Exos enhance autophagy and then slow the progression of DN via the mTOR signalling pathway [88]. Jin et al. further showed that the ADMSC-Exo can inhibit the Smad1/mTOR signalling pathway by miRNA-486, which promotes autophagy and inhibits apoptosis in podocytes, thus ameliorating the symptoms of DN [89]. Details of the aforementioned trials are summarised in Table 2.

**Table 2:** Functional pathways of MSC-EVs in DN models

Model	Animal	In vitro model	Injection	EVs/MVs/EXO of source	Pathway/key substance	Target	Mechanism	Reference
STZ	SD rat	HPDCs	Tail vein	hUSC-Exo	miRNA-16-5p	VEGFA podocytes	Anti-apoptosis	[83]
	C57BL/KsJ db/m mouse	MP5 cell	Tail vein	ADMSC-EVs	miRNA-26a-5p	TLR4	Anti-apoptosis	[84]
STZ	Babl/c mouse	HK-2 cell	Tail vein	huMSC–MVs	miRNA-451a	P15 and P19	Anti-fibrosis	[85]
STZ	NSG mouse		Tail vein	HLSC/MSC EVs	Serpina1a, FAS ligand, CCL3, TIMP1, MMP3, type I collagen, and Snail		Anti-fibrosis	[86]
		MPC5 cell		ADMSC-Exo	miRNA-215-5p	ZEB2	Anti-fibrosis	[87]
STZ	Albino rat		Tail vein	BMMSC-Exo		mTOR pathway	Inducing autophagy	[88]
		MPC5 cell		ADMSC-Exo	miRNA-486	Smad1/mTOR signalling pathway	Pro-autophagy	[89]

*MSCs* mesenchymal stem cells, *EVs* extracellular vesicles, *DN* diabetic nephropathy, *STZ* streptozotocin

#### MSC-EVs and atherosclerotic renovascular diseases

Atherosclerosis is the primary cause of renal artery stenosis. Atherosclerotic renovascular disease (ARVD) can induce chronic renal ischaemia and further lead to fibrosis, which develops to ESRD. Percutaneous transluminal renal angioplasty is a common surgery for treating ARVD; however, it is difficult to restore functions of the atrophic kidney. Animal experiments have confirmed that the combination of MSCs with ARVD to treat atherosclerotic renal artery stenosis helps to restore functions of the kidney [90]. Thereafter, several clinical trials have evinced the safety of infusing autologous ADMSCs in the treatment of ARVD [91–93]. Following this, ADMSC-EVs have also become the focus of recent research. In the model of unilateral renovascular disease complicating metabolic syndrome (MetS), Eirin et al. proved that the autologous ADMSC-EVs improve the renal microvascular system in pigs with metabolic renal vascular diseases [94]. Besides this, Simeoni et al. further identified the miRNA in MSC-EVs as an important target for ARVD [95]. In addition, MSC-EVs were also found to enhance the advantages of Treg by TGF-β therein and therefore improve the functions of the kidney with renal artery stenosis in the MetS+RAS model [96]. Autologous ADMSC-EVs can also prompt the transformation of phenotypes of macrophages from M1 to M2 via IL-10, so as to relieve renal artery stenosis [97].

At the same time, some researchers proposed that MSC-Exos can only partially relieve ageing kidney induced by renal artery stenosis [98]. MetS is able to change the amount of loading of miRNA on EVs, upregulate ageing-related miRNA in EVs, and even limit the use of EVs in exogenous regenerative therapy through abnormal transcription [99–101]. Zhao et al. found that autologous ADMSCs can better preserve microcirculation through comparative studies, while ADMSC-EVs perform better in retaining intactness of nephrocytes and reducing necrosis [102]. In summary, the application value of MSC-EVs in the treatment of ARVD remains in dispute, and further research is warranted to reveal their efficacy.

### MSC-EVs and kidney transplantation

Kidney transplantation is the preferred treatment method for end-stage renal failure patients. The shortage of donor organs and the half-life of the transplant limit the therapy [4]. In addition, ischaemia-induced AKI is widely seen in kidney transplantation due to the time available for the development of ischaemia given the delay between the accession of the kidney from the donor to renal ischaemia reperfusion in receptors [103]. This is also a major cause of the delayed functions of the transplant. To solve these problems, static cold storage, hypothermic machine perfusion (HMP), and several new drug candidates targeting ischaemia and reperfusion are under study [104]. The work of del Rio et al. verified that HMP and normothermic regional perfusion are preferable to static cold storage [105]. Also, researchers are devoted to find other effective ways to complement the current techniques.

A trial involving 105 Chinese kidney transplantation subjects who received autologous MSCs in the reperfusion of the transplanted kidney suggests that it is feasible and safe to use MSCs in kidney transplantation [106]; however, a similar trial conducted by another research team recently found that the post-operative complications of renal transplantation, infectious complications, kidney functions, rejection frequency, and survival time all do not show statistical differences with the control in the 1-year follow-up [107]. Therefore, the protective effect of MSC-EVs in transplanted kidney remains a matter of dispute. Gregorini et al. proved that adding MSCs/EVs to the Belzer solution in the HMP period can protect the kidney from ischaemic injury by preserving the enzymatic mechanism essential to cell viability [108]. Experiments by Koch et al. indicated that MSC-EVs regulate the immunoreaction to allogeneic kidney transplants to some extent [109]. Significantly different from this, by establishing a rat model of heterotopic kidney transplantation, Jose Ramirez-Bajo et al. found that autologous MSCs prolong the survival time of transplants and subjects in the rat model of renal rejection, while EVs do not [110]. This topic is rarely studied, and further research is required before a conclusion can be drawn.

## Problems and prospects

In previous research, MSCs have been found to play their positive roles in treating various kidney diseases. For example, MSC-CM relieves the experimental anti-glomerular basement membrane glomerulonephritis by virtue of the M2 macrophage-mediated anti-inflammatory action [111]. In systemic lupus erythematosus (SLE), allogeneic MSC transplantation mitigates kidney injury [112]. Clinical trials also show that it is both safe and feasible to treat SLE patients with allogeneic MSCs from healthy donors [113]. In the model of nephrotic syndrome induced by Adriamycin, MSCs mainly play their role in kidney repair by regulating inflammation [114]. Moreover, healthy donors and idiopathic nephrotic syndrome (INS) patients do not exhibit obvious differences in the functions and morphologies of MSCs, which indicates that MSCs can be used for treating INS with autologous cells [115]. MSC treatment exerts beneficial effects on IgAN by the mechanism of paracrine that modulates the balance of the Th1/Th2 cytokine [116]. In the rat model of anti-Thy1.1-induced glomerulonephritis, hypoxic-preconditioned MSCs decrease glomerular apoptosis, autophagy, and inflammation through signal transduction of HIF1α/VEGF/Nrf2 [117]. MSCs relieve renal hypertension and improve kidney function in the 2-kidney, 1-clip model [118]. No adverse events and severe adverse events were observed clinically when treating anti-body against antineutrophil cytoplasmic antibody-associated vasculitis [119] and autosomal dominant polycystic kidney disease [120] with autologous mesenchymal stromal cells. Existing research also proposes that MSCs possibly relieve focal segmental glomerulosclerosis via IL-22 [121].

In conclusion, both animal models and clinical trials provided much evidence of the potential of MSCs in the treatment of kidney diseases; however, there is little research into the treatment of the aforementioned diseases with MSC-EVs, which remains to be explored. This is possibly because the separation, purification, and mass production of EVs remain a challenge; moreover, the mechanism by which MSC-EVs treat kidney diseases has not been elucidated. In addition, in consideration of optimal source, appropriate dosage, and appropriate route of administration of EVs, further research needs to be undertaken to assess the efficacy of the application of MSC-EVs to clinical treatment of kidney diseases.

## Conclusion

In this review, we summarised the recent advances of complex and critical effects of MSC-EVs in kidney diseases, including AKI, CKD, DN, ARVD, and kidney transplantation. A large number of articles support that most kidney diseases can benefit from MSC-EVs; however, the effects of kidney transplantation are still controversial. Although MSC-EVs isolated from different sources show great promise as therapeutic agents for kidney diseases in animal studies and preclinical trials, further studies are necessary since only few clinical works have been described at the moment.

## Data Availability

The datasets used and analysed in the present research are available from the corresponding author upon reasonable request.

## Abbreviations

MSCs: Mesenchymal stem cells
EVs: Extracellular vesicles
MSC-EVs: MSC-derived EVs
CKD: Chronic kidney disease
DN: Diabetic nephropathy
ARVD: Atherosclerotic renovascular disease
AKI: Acute kidney injury
BMMSCs: Bone marrow-derived MSCs
ADMSCs: Adipose-derived MSCs
huMSCs: Human umbilical cord-derived MSCs
mRNA: Messenger ribose nucleic acid
miRNA: Micro-ribose nucleic acid
MVs: Microvesicles
Exos: Exosomes
MVBs: Multivesicular bodies
RGD: Arginine-glycine-aspartate
IRI: Ischaemic reperfusion injury
I/R: Ischaemia-reperfusion
CLP: Caecal ligation and puncture
hWJMSCs: Human Wharton’s jelly MSCs
hWJMSC-EVs: EVs derived from hWJMSCs
TECs: Tubular epithelial cells
ERK: Extracellular regulated kinase
UUO: Unilateral ureteral obstruction
HLSC: Human liver stem cell
ARVD: Atherosclerotic renovascular disease
MetS: Metabolic syndrome
HMP: Hypothermic machine perfusion
SLE: Systemic lupus erythematosus
INS: Idiopathic nephrotic syndrome
